# Muscle Mass Mediates the Effect of Physical Activity and Sedentary Behavior on Metabolic Syndrome, with Differences by Gender

**DOI:** 10.3390/healthcare13192432

**Published:** 2025-09-25

**Authors:** Jaehee Kim

**Affiliations:** Graduate School of Alternative Medicine, Kyonggi University (Seoul Campus), 24, Kyonggidae-ro 9-gil, Seodaemun-gu, Seoul 03746, Republic of Korea; jk41@kyonggi.ac.kr

**Keywords:** cardiometabolic health, metabolic syndrome, physical activity, sedentary behavior, skeletal muscle mass

## Abstract

Objectives: This study examined whether skeletal muscle mass mediates the relationship between sedentary behavior, physical activity, and cardiometabolic health, and if this relationship differs by genders. Methods: Secondary analysis was conducted using data from the 2022–2023 Korean National Health and Nutrition Examination Survey (*n* = 5956). Cardiometabolic abnormalities were defined as having one or more of the five metabolic syndrome (MS) criteria, while MS was defined as having three or more. Muscle mass relative to body weight was measured by bioelectrical impedance analysis. Sedentary time and recommended moderate-to-vigorous physical activity (MVPA) levels at work, for transportation, and for recreation (≥600 Mets), and strength training (≥2 times/week), were assessed using the Global Physical Activity Questionnaire. Results: Higher MVPA (*p* < 0.001) and less sedentary time (*p* < 0.01) were significantly correlated with greater muscle mass in middle-aged and elderly men and women. Mediation analyses, which controlled for MS risk factors, revealed gender differences. In men, the indirect effects of sedentary time, MVPA, and strength training on both cardiometabolic abnormalities (*b* = 0.007, CI [0.003, 0.013]; *b* = −0.066, CI [−0.110, −0.033]; *b* = −0.074, CI [−0.110, −0.033]) and MS (*b* = 0.007, CI [0.003, 0.011]; *b* = −0.060, CI [−0.095, −0.032]; *b* = −0.065, CI [−0.100, −0.035]) were significant, indicating mediation by muscle mass. In women, the indirect effects of these three behaviors on cardiometabolic abnormalities were also mediated by muscle mass (*b* = 0.003, CI [0.001, 0.006]; *b* = −0.014, CI [−0.031, −0.002]; *b* = −0.023, CI [−0.050, −0.003]). However, for MS, sedentary time (*b* = 0.057, *p* < 0.001) and MVPA (*b* = −0.222, *p* < 0.05) had only direct effects, with no mediation by muscle mass. Furthermore, strength training showed no significant effects. Conclusions: These findings suggest that promoting MVPA and strength training, while reducing sedentary time, can improve cardiometabolic health by increasing muscle mass, though the mediating role of muscle mass for MS differs by gender.

## 1. Introduction

Metabolic syndrome (MS) is defined as a cluster of at least three cardiometabolic abnormalities, including hyperglycemia, abdominal obesity, dyslipidemia, and elevated blood pressure [1]. Having MS significantly increases the risk for cardiovascular disease, diabetes, and all-cause mortality [1,2,3]. Even one or two of these cardiometabolic abnormalities can raise the risk for all-cause mortality [2], and so managing them is crucial for public health.

Known risk factors for MS include biological factors like age, gender, menopause, family history, as well as sociodemographic factors such as income and education. Lifestyle choices also play a significant role [4,5,6,7,8], particularly physical inactivity and sedentary behavior, which are considered the main modifiable risk factors [8,9,10]. Conversely, following physical activity guidelines—including at least 600 MET-minutes per week of moderate-to-vigorous physical activity (MVPA) and two or more days of strength training, along with reduced sedentary time—can help reduce the risk of developing metabolic syndrome [8,10,11,12,13,14].

The underlying mechanisms linking sedentary time and a lack of physical activity to cardiometabolic health are complex, but they likely involve skeletal muscle [15,16]. Skeletal muscle is crucial for managing cardiometabolic health because it helps regulate lipid metabolism, glucose uptake, and insulin sensitivity [17,18,19,20]. Reduced skeletal muscle mass is a known risk factor for MS [21,22,23]. Sedentary behavior is associated with lower muscle mass, while physical activity, including MVPA and strength training, is known to increase it [14,24,25]. Therefore, skeletal muscle mass likely acts as a mediator, helping to explain the impact of sedentary time and physical activity on cardiometabolic health.

No study has specifically investigated whether skeletal muscle mass mediates the relationship between sedentary time, physical activity, and cardiometabolic health. A mediation analysis, as used in this study, could determine if increasing physical activity and decreasing sedentary time improve cardiometabolic health by impacting skeletal muscle mass. Providing a more integrated understanding of these complex interactions, study findings can help create better strategies and practical lifestyle guidelines for preventing metabolic syndrome, including recommendations for reducing sedentary time and increasing physical activity to maintain muscle mass.

The purpose of this study was to investigate if skeletal muscle mass mediates the influence of sedentary time and physical activity—including MVPA and strength training—on cardiometabolic abnormalities and MS in middle-aged and elderly Korean men and women, after adjusting for other risk factors. I hypothesize that meeting recommended physical activity levels and decreasing sedentary time increases muscle mass, which in turn decreases the risk of being cardiometabolically abnormal. Additionally, gender differences were examined based on these hypotheses.

Accordingly, for this analysis, having MS and one or more cardiometabolic abnormalities served as the dependent variables. The independent variables included weekly MVPA (≥600 METs), strength training (≥2 times/week), and daily sedentary time. Muscle mass was used as a mediating variable, and MS risk factors were controlled as covariates in the mediation analysis.

## 2. Materials and Methods

### 2.1. Study Design

Secondary analysis was performed using data from the Korean National Health and Nutrition Examination Survey (KNHANES), conducted by the Korea Disease Control and Prevention Agency. In brief, the KNHANES is conducted annually using a rolling sampling design. The KNHANES data, a nationally representative cross-sectional sample, used sampling units, defined by geographic area, gender, and age group, from the household registries of the National Census Registry in South Korea [26].

Data from the 2022–2023 KNHANES were selected because they included all measurements for the independent variables (physical activity and sedentary behavior), the dependent variable (MS), and the mediator (skeletal muscle mass). Physical activity and sedentary behavior were assessed by the Global Physical Activity Questionnaire (GPAQ), and skeletal muscle mass was measured using bioelectrical impedance analysis (BIA).

The protocol of KNHANES was approved by Institutional Review Boards of the Korea Disease Control and Prevention Agency (IRB no. 2018-01-03-4C-A for 2022 and 2022-11 16-R-A for 2023). All participants gave written informed consent.

### 2.2. Study Participants

A total of 8655 middle-aged and elderly men and women aged 40 to 80 years were initially included from the 2022–2023 KNHANES data. A total of 2699 participants were excluded for the following reasons: missing values on independent and dependent variables, the mediator, and covariates; fasting for less than 8 h; being pregnant; or being underweight due to the extremely low prevalence of metabolic syndrome in the study sample. Finally, 5956 participants were included in the data analysis.

### 2.3. Definition of Metabolic Syndrome and Cardiometabolic Abnormalities

For the definition of MS, the National Cholesterol Education Program Adult Treatment Panel III criteria were used [1]. A subject was classified as having metabolic syndrome if they met at least three of the five following criteria: systolic blood pressure ≥ 130 mmHg, diastolic blood pressure ≥ 85 mmHg, or currently taking antihypertensive medication; fasting plasma glucose ≥ 100 mg/dL or currently taking antidiabetic medication; triglyceride ≥ 150 mg/dL or currently taking medication for dyslipidemia; high-density lipoprotein (HDL) cholesterol < 40 mg/dL in men and <50 mg/dL in women, or currently taking medication for dyslipidemia; waist circumference ≥ 90 cm in men and ≥85 cm in women, based on criteria from the Korean Society of Obesity [27]. Having a cardiometabolic abnormality was defined as meeting one or more of these criteria.

### 2.4. Skeletal Muscle Mass

Skeletal muscle mass was measured by a simultaneous multi-frequency BIA machine (InBody 970, Biospace, Seoul, Republic of Korea), equipped with a grab lead and a tetrapolar 8-point tactile electrode system. This machine measures the impedance of five body segments (the torso, both arms, and both legs) at frequencies of 5, 50, 250, 500 kHz, 1 MHz, and 3 MHz [28].

Participants fasted for at least 8 h before the measurements. To prepare for the test, participants wore light clothing, removed all metal components, and rested in a standing position for a minimum of 10 min. Participants stood barefoot on the foot electrodes while holding the hand electrodes. They were instructed to extend their arms straight out to prevent contact between the armpits and the body and avoid thighs touching. Participants wrapped all four fingers around the hand electrodes, with their thumbs placed on the designated thumb electrodes, and aligned their heels with the end of the foot electrodes.

Skeletal muscle mass was defined as the appendicular skeletal muscle mass, calculated as the sum of lean soft tissue in both arms and legs, and then normalized as percentage of body weight. This weight-adjusted index has been widely used alongside the height-squared and BMI-adjusted indices. Among these three, there is no clear consensus on the most appropriate one for determining muscle mass [29]. Appendicular lean soft tissue measured by dual-energy X-ray absorptiometry is known to correlate well with total-body skeletal muscle mass measured by whole-body magnetic resonance imaging [30]. The appendicular lean soft tissue measured by the BIA machine used in this study also showed a high correlation with the values from dual-energy X-ray absorptiometry [31].

### 2.5. Sedentary Behavior and Physical Activity

The MVPA, strength training, and sedentary behavior were assessed using the Korean version of the GPAQ [13,26], an instrument demonstrated to be both reliable and valid [32,33]. The GPAQ gathers information on the days and time spent engaging in activities at work, during recreation, and for transportation (e.g., walking or cycling) in a typical week.

Physical activity levels were then converted to metabolic equivalent of task (MET) values: 4 METs for moderate activities and transportation, and 8 METs for vigorous activities, in accordance with the GPAQ scoring protocol [13]. After calculating the MET-minutes per week for each activity across the domains of work, recreation, and transportation, these values were summed to obtain the total MET-minutes per week.

**Figure 1:**
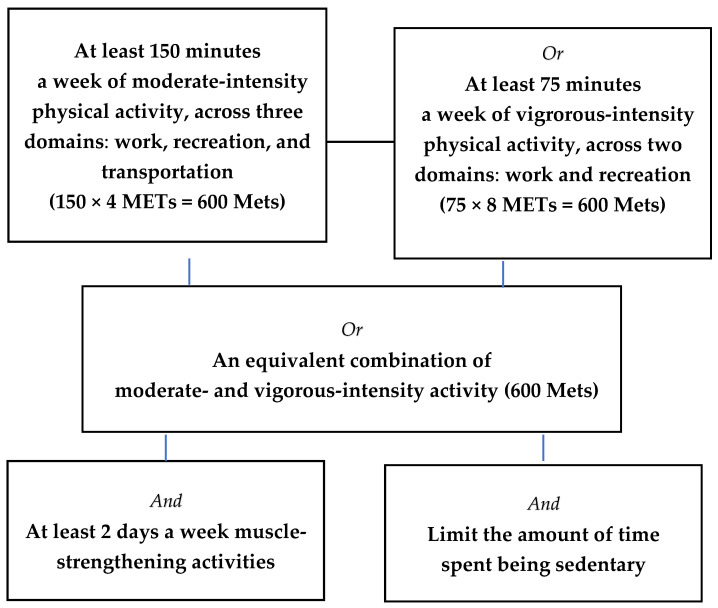
Recommend physical activity guidelines for optimal health benefits in adult and elderly men and women.

Recommend physical activity guidelines for optimal health benefits by WHO in adult and elderly men and women are presented in Figure 1 [14]. Weekly MVPA was subsequently categorized as either ≥600 MET-minutes or <600 MET-minutes according to WHO recommendations for optimal health benefits, which suggest at least 600 MET-minutes per week through a combination of moderate and vigorous physical activities [13]. Strength training was defined as being performed 2 or more days per week [14]. Sedentary behavior was assessed by daily sitting time in hours.

### 2.6. Risk Factors for Metabolic Syndrome as Covariates

Sociodemographic factors and known risk factors associated with metabolic syndrome were included in the mediation analysis as covariates: age (years), body mass index (BMI, kg/m^2^), education levels, marital status, household income, family history of chronic diseases (hypertension, dyslipidemia, ischemic heart disease, stroke, and diabetes), menopausal status for women, smoking status, alcohol consumption, daily total energy intake (kcal), and the proportion of daily energy (%) from protein, fat, and carbohydrate [5,6,7].

Sociodemographic factors, health condition, and dietary habit were assessed using questionnaires during a face-to-face interview in a mobile examination vehicle. Smoking and drinking habits were self-reported. Weight and height were measured to calculate BMI.

The 24 h dietary recall method was used to collect dietary information. The food intake questionnaire was an open-ended survey designed for participants to report the various dishes and foods they consumed, along with the frequency and quantity. Participants were asked to recall and report everything they ate and drank over the previous two days. This recall was conducted two days before the survey to account for the fasting time required before the blood test. The raw nutritional data from the KNHANES provided the amount of each nutrient intake. To calculate the energy intake ratio, the intake of carbohydrates was multiplied by 4 kcal, protein by 4 kcal, and fat by 9 kcal. Each value was then divided by the total daily energy intake and multiplied by 100 to obtain the percentage.

### 2.7. Statistical Analysis

The data were analyzed using SPSS version 29.0 (IBM SPSS Statistics, Armonk, NY, USA). The significance level for all analyses was set at *p* < 0.05. Independent *t*-tests for continuous variables and Chi-square tests for categorical variables were used to assess group differences in participants’ characteristics between the non-MS and MS groups. Correlations among age, BMI, skeletal muscle mass, sedentary time, weekly total MVPA, and cardiometabolic variables were assessed using Pearson correlation coefficients.

A Simple logistic regression analysis was conducted to identify risk factors for MS as covariates. Variables with a statistically significant odds ratio (OR) were included as covariates in the mediation model. The tested variables were: age, BMI, education levels (0 = high school graduate or less, 1 = a college education or higher), marital status (0 = married, 1 = unmarried), household income (0 = median or above, 1 = below median), family history of chronic diseases (0 = not having or unknown, 1 = presence of one or more disease), menopausal status (0 = premenopausal, 1 = postmenopausal), smoking status (0 = non-smoker, 1 = past smoker or current smoker), alcohol consumption (0 = non-drinker or less than 1 drink per month, 1 = 1 drink or more per month), total energy intake, protein intake, fat intake, and carbohydrate intake.

**Figure 2:**
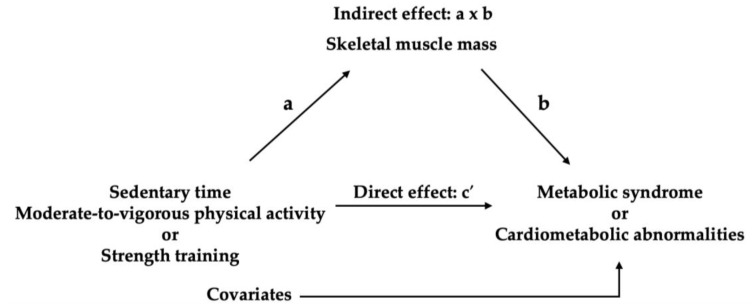
Mediation model.

Mediation analyses (model 4) with 5000 bootstrap samples were conducted controlling for covariates using the PROCESS macro (version 5.0) for SPSS. These analyses aimed to test whether effect of independent variables (X) on dependent variables (Y) is mediated by mediator (M) [34]. A schematic diagram of the mediation model is presented in Figure 2. The PROCESS macro estimates the direct effect (c’) of X (e.g., sedentary time, MVPA, and strength training) on Y (e.g., cardiometabolic abnormalities and MS) after accounting for M (skeletal muscle mass) and the indirect effect (a x b) through M. A covariate-adjusted direct and indirect effects were presented as regression coefficients (*b*). An indirect effect is considered significant if the 95% confidence interval (CI) of its bootstrapped estimate does not include zero, indicating that mediation has occurred [34].

## 3. Results

### 3.1. Participant Characteristics by Gender

The prevalence of MS was significantly higher in middle-aged and elderly men (39.9%) compared to women (26.7%, *p* < 0.001). Table 1 presents the characteristics of the 2568 male and 3388 female participants. In both genders, compared to the non-MS group, the MS group exhibited higher values in anthropometric and cardiometabolic variables, including BMI, waist circumference, systolic and diastolic blood pressures, glucose, and triglycerides (all *p* < 0.001). This group also had lower HDL cholesterol and a lower skeletal muscle mass (all *p* < 0.001).

Regarding health and socioeconomic factors, a family history of chronic disease was more prevalent in men with MS (*p* < 0.001) but not in women. Additionally, a higher proportion of women with MS were postmenopausal (*p* < 0.001). Conversely, individuals without MS were more likely to be married, have a college education or higher, and have a monthly household income above the median. These differences were significant in both men (*p* < 0.05 or 0.01) and women (all *p* < 0.001).

Lifestyle behaviors also varied between the groups. Men with MS were more likely to be smokers (*p* < 0.01), though there were no differences in drinking and sedentary time. In women, the non-MS group showed a higher proportion of drinkers and lower mean sedentary time (both *p* < 0.001) with no difference in smoking. Furthermore, in both men and women, the MS group engaged in less physical activity, as indicated by lower weekly MVPA levels (MET-minutes), fewer participants achieving 600 MET-minutes of MVPA or more, and a lower proportion engaging in strength training more than two days per week (*p* < 0.01 or *p* < 0.001).

Dietary intake patterns also differed by gender and MS status. Men with MS had a higher mean total daily energy intake compared to the non-MS group (*p* < 0.05), with no differences in carbohydrate, fat, or protein intake. In contrast, women with MS showed a higher intake of carbohydrate but lower intakes of total energy, fat, and protein compared to the non-MS group (all *p* < 0.001).

### 3.2. Correlations Among Skeletal Muiscle Mass, Sedentary Time, MVPA, and Cardiometabolic Variables

The Pearson correlation coefficients among age (years), BMI (kg/m^2^), skeletal muscle mass (%), the five MS criteria, the total number of MS criteria, sedentary time (hours/day), and weekly MVPA levels (MET-minutes per week) in all participants (*n* = 5956) are presented in Table 2. Main findings are as follow: Skeletal muscle mass significantly decreased with increasing age and BMI (both *p* < 0.001). Conversely, greater skeletal muscle mass was significantly associated with a higher level of MVPA (*p* < 0.001) and less sedentary time (*p* < 0.01).

The total number of MS criteria increased significantly with increasing age, BMI, and sedentary time, while decreased with higher MVPA levels and greater skeletal muscle mass (all *p* < 0.001). Specifically, longer sedentary time was significantly associated with a greater waist circumference, higher triglycerides levels, and lower HDL-cholesterol levels (all *p* < 0.001). In contrast, a higher level of MVPA was linked to improved cardiometabolic variables, including lower systolic blood pressure (*p* < 0.05), lower glucose levels (*p* < 0.01), and higher HDL-cholesterol levels (*p* < 0.001). Furthermore, higher levels of MVPA were correlated with lower sedentary time (*p* < 0.001), suggesting an inverse relationship between these two behaviors.

### 3.3. Simple Effects of Independent Variables, a Mediator, and Covariates on Metabolic Syndrome

Simple logistic regression analyses were performed to identify covariates, with presence of MS serving as the dependent variable (*n* = 5956). The variables tested included age, BMI, education levels, marital status, household income, family history of chronic diseases, menopausal status, smoking status, alcohol consumption, daily total energy intake, and the proportion of daily energy from protein, fat, and carbohydrate.

Unadjusted ORs for MS significantly increased with increasing age (OR 1.03, CI 1.02–1.03, *p* < 0.001) and BMI (OR 1.42, CI 1.39–1.45, *p* < 0.001), unmarried status (OR 1.38, CI 1.21–1.57, *p* < 0.001), smokers (OR 1.65, CI 1.43–1.91, *p* < 0.001), higher daily energy intake (OR 1.00, CI 1.00–1.00, *p* < 0.01), and higher carbohydrate intake (OR 1.01, CI 1.00–1.01, *p* < 0.001). Conversely, unadjusted ORs for MS significantly decreased with a college education or higher (OR 0.59, CI 0.53–0.67, *p* < 0.001), monthly household income above the median (OR 0.68, CI 0.61–0.76, *p* < 0.001), higher protein intake (OR 0.97, CI 0.96–0.99, *p* < 0.001), and higher fat intake (OR 0.97, CI 0.97–0.98, *p* < 0.001). In women (*n* = 3388), unadjusted ORs for MS significantly increased with menopausal status (OR 2.12, CI 1.68–2.67, *p* < 0.001). Family history of chronic diseases and alcohol consumption were not significant factors for MS. Therefore, these two variables were excluded from covariates.

Regarding effects of independent variables and mediator on MS, unadjusted ORs for MS significantly increased with longer sedentary time (OR 1.05, CI 1.03–1.06, *p* < 0.001) and decreased with achieving 600 MET-minutes of MVPA or more (OR 0.71, CI 0.63–0.79, *p* < 0.001), engaging in strength training 2 or more days per week (OR 0.75, CI 0.65–0.85, *p* < 0.001), and greater skeletal muscle mass relative to body weight (OR 0.91, CI 0.90–0.93, *p* < 0.001).

### 3.4. Direct and Indirect Effects of Sedentary Time and Physical Activity on Cardiometabolic Abnormalities and Metabolic Syndrome, Mediated by Skeletal Muscle Mass

Results of the mediation analyses are presented in Table 3. Mediation analyses were conducted to examine the direct effects of independent variables and their indirect effects, mediated by skeletal muscle mass, on cardiometabolic abnormalities and MS. Covariates were controlled for in the analyses. The independent variables included sedentary time (hours per day), engaging in MVPA (0 = less than 600 MET-minutes per week, 1 = 600 MET-minutes or more), and strength training (0 = less than 2 days per week, 1 = 2 days or more). Cardiometabolic abnormalities were defined as a binary outcome (0 = No, 1 = presence of one or more MS criteria). MS was also defined as a binary outcome (0 = presence of fewer than three MS criteria, 1 = presence of three or more MS criteria). An indirect effect was considered statistically significant if its 95% CI did not include zero.

In men, a significant indirect effect of sedentary time (*b* = 0.007, CI [0.003, 0.013]), MVPA (*b* = −0.066, CI [−0.110, −0.033]), and strength training (*b* = −0.074, CI [−0.110, −0.033]) on cardiometabolic abnormalities was observed through skeletal muscle mass, even after controlling for covariates. The direct effects of these three independent variables were not significant, indicating that skeletal muscle mass fully mediated the relationships between these activities and cardiometabolic abnormalities. This finding suggests that greater sitting time was associated with decreased skeletal muscle mass, which in turn led to an increase in cardiometabolic abnormalities. Conversely, engaging in MVPA and strength training likely reduce cardiometabolic abnormalities by augmenting skeletal muscle mass.

Furthermore, in men, mediation analyses employing skeletal muscle mass as a mediator and MS as the dependent variable revealed that sedentary time and MVPA had no direct effects on MS. Instead, their effects were fully mediated by skeletal muscle mass. The indirect effects of sedentary time (*b* = 0.007, CI [0.003, 0.011]) and MVPA (*b* = −0.060, CI [−0.095, −0.032]) on MS through skeletal muscle mass were significant. This suggests that sedentary time indirectly increased the MS by decreasing skeletal muscle mass, while MVPA indirectly decreased it by increasing skeletal muscle mass. In contrast, strength training demonstrated both a direct effect (*b* = −0.399, *p* < 0.001) on MS and an indirect effect via increased skeletal muscle mass (*b* = −0.065, CI [−0.100, −0.035]).

In women, sedentary time and MVPA did not directly affect cardiometabolic abnormalities, after controlling for covariates. Instead, the indirect effects of sedentary time (*b* = 0.003, CI [0.001, 0.006]) and MVPA (*b* = −0.014, CI [−0.031, −0.002]) on cardiometabolic abnormalities through skeletal muscle mass were significant. Their impacts were fully mediated by skeletal muscle mass. This suggests that prolonged sedentary time indirectly raised cardiometabolic abnormalities by decreasing skeletal muscle mass, while MVPA indirectly decreased it by reducing skeletal muscle mass. In contrast, strength training showed both a direct effect (*b* = −0.346, *p* < 0.01) on cardiometabolic abnormalities and an indirect effect through skeletal muscle mass (*b* = −0.023, CI [−0.050, −0.003]).

Furthermore, in women, no indirect effects of sedentary time, MVPA, or strength training through skeletal muscle mass on MS were observed. Instead, significant direct effects were found for sedentary time (*b* = 0.057, *p* < 0.001) and MVPA (*b* = −0.222, *p* < 0.05) and not for strength training. This indicates that sedentary time directly increases MS, and MVPA directly decreases it, while skeletal muscle mass did not significantly mediate these relationships. Strength training, in this context, demonstrated neither a direct nor an indirect effect on MS through skeletal muscle mass.

## 4. Discussion

This study investigated the mediating effect of skeletal muscle mass on the associations of sedentary behavior, MVPA, and strength training with cardiometabolic abnormalities and MS in middle-aged and elderly Korean men and women. The findings revealed gender differences in the mediating role of skeletal muscle mass.

In men, the effects of sedentary time and MVPA on the likelihood of having one or more cardiometabolic abnormalities or MS were fully mediated by skeletal muscle mass. This means their impact was entirely indirect. Specifically, more sedentary time and not achieving 600 or more MET-minutes of MVPA per week led to decreased muscle mass, which in turn increased the likelihood of having cardiometabolic abnormalities and MS. Furthermore, the effect of strength training on cardiometabolic abnormalities was also completely mediated by skeletal muscle mass. Performing strength training at least two days a week reduces the likelihood of having cardiometabolic abnormalities through an increase in skeletal muscle mass. For MS, however, the effect was only partially mediated. This suggests a dual benefit: strength training indirectly reduces the likelihood of having MS by increasing muscle mass, and it also has an independent, direct effect.

The results for women show both similarities and differences when compared to men. The full mediation of muscle mass on the effect of sedentary time and MVPA on cardiometabolic abnormalities was observed consistently with the findings in men. In women, strength training has both a direct and an indirect effect on reducing the likelihood of having cardiometabolic abnormalities. This suggests that muscle mass is an equally vital link for both genders in preventing cardiometabolic abnormalities. In women, unlike in men, strength training also has a direct effect that is independent of muscle mass.

However, when it comes to MS, the findings are different. For women, the results show no mediating role of skeletal muscle mass in the relationship between sedentary time, MVPA, or strength training and MS. Instead, sedentary time and MVPA have direct effects on MS. This suggests that in women, they influence the likelihood of having MS through pathways that are not dependent on changes in muscle mass. The lack of both direct and indirect effects of strength training on MS in women is also a noteworthy finding that warrants further investigation.

Previous studies on the effects of sedentary time and physical activity on cardiometabolic health have focused on their relationships. High levels of total sedentary time have been significantly associated with an increased likelihood of being MS [8,9]. Conversely, achieving at least 600 MET-minutes of MVPA per week is significantly associated with a lower likelihood of being MS [10,12].

While no studies have specifically examined the indirect effect of sedentary behavior and physical activity on the cardiometabolic health as mediated by skeletal muscle mass, previous studies have reported a significant association between these factors. Sedentary time is associated with lower skeletal muscle mass, whereas performing MVPA and strength training are known to increase it [15,24,25]. Furthermore, reduced skeletal muscle mass is associated with insulin resistance, prediabetes, and MS [21,22]. Altogether, these findings suggest a potential mediational pathway where physical activity and sedentary time impact cardiometabolic health through their effects on skeletal muscle.

Consistent with previous studies, in this study, correlation analyses revealed that a lower level of MVPA and longer sedentary time were significantly associated with less skeletal muscle mass [24,35]. Furthermore, individuals who engaged in more MVPA were less sedentary. Additionally, longer sedentary time, lower weekly MVPA, and less muscle mass were correlated with a higher number of MS criteria, suggesting having more cardiometabolic abnormalities. Notably, results from mediation analyses indicate that less sedentary time and meeting weekly recommended MVPA and strength training improve cardiometabolic health by increasing muscle mass.

Having cardiometabolic abnormalities, including MS, is related to insulin resistance, dyslipidemia, inflammation, and oxidative stress [36,37,38]. Prolonged sedentary time and lack of physical activity may exacerbate cardiometabolic health by influencing these mechanisms. One of the underlying mechanisms by which sedentary time and physical activity affect cardiometabolic health involves changes in skeletal muscle [15,16]. Physical inactivity increases intramuscular fat infiltration, which in turn causes insulin resistance and dyslipidemia [39].

Skeletal muscle influences cardiometabolic health through several interconnected mechanisms. As the largest site of insulin-mediated glucose uptake, skeletal muscle plays a key role in glycemic regulation and lipid metabolism [17,18,19,20]. Loss of skeletal muscle mass is associated with increased insulin resistance [18]. A previous study reported that disruption of glucose transporter 4 selectively in skeletal muscle resulted in severe insulin resistance and glucose intolerance, suggesting that muscle is crucial for the normal glucose homeostasis [40]. Skeletal muscle also acts as an endocrine organ, secreting myokines such as interleukin-6 (IL-6) and irisin [18,19]. Exercise-induced muscle IL-6 stimulates the anti-inflammatory cytokines IL-1ra and IL-10, inhibits the production of the pro-inflammatory cytokines such as tumor necrosis factor-α, and promotes lipolysis as well as fat oxidation [41]. Irisin, which increases energy expenditure and improves insulin sensitivity, is positively associated with physical activity and skeletal muscle mass [18,42]. Therefore, having more muscle mass could be beneficial for overall cardiometabolic health.

In this study, the significant direct effects of physical activity and sedentary time were observed after controlling for muscle mass and covariates, suggesting an independent biological mechanism not solely dependent on muscle size. This could be related to improvements in insulin sensitivity, a reduction in low-grade chronic inflammation, and an increase in the resistance against oxidative stress [41,43,44].

For women, the results show no mediating role of skeletal muscle mass in the relationship between sedentary time, MVPA, or strength training and metabolic syndrome. Instead, sedentary time and MVPA have significant direct effects on the likelihood of having MS. These findings may imply that different mechanisms exist between genders for developing metabolic syndrome. The participants in this study were middle-aged and elderly adults. The difference from men could be due to hormonal differences, such as the loss of estrogen due to menopause [45].

This study has a key strength: While previous studies focused on the individual effects of physical activity, sedentary behavior, and muscle mass, this study integrates these variables and accounts for confounding factors. This approach contributes to a more comprehensive understanding of the complex interactions among multiple risk factors, which can lead to more effective strategies for managing cardiometabolic health. Additionally, the study’s use of a large, nationally representative dataset further strengthens its findings.

Physical inactivity and sedentary behavior can lead to sarcopenia, a progressive loss of muscle mass that increases the risk of cardiometabolic diseases [46]. This highlights the importance of muscle-centric interventions, such as MVPA and strength training, as a primary strategy for enhancing metabolic health, especially in older adults. Rather than viewing muscle mass merely as a health indicator, this study reframes muscle preservation as a central therapeutic target—a strategic and modifiable mechanism for mitigating age-related cardiometabolic abnormalities. This therapeutic approach also empowers older adults to proactively combat sarcopenia, a condition often overlooked by traditional screening methods like BMI because of the prevalence of sarcopenic obesity [46].

### Limitations of the Study and Future Recommendations

This study also had some limitations. First, although mediation analyses were performed, its cross-sectional design prevents a strong causal relationship from being established between physical activity, muscle mass, and cardiometabolic health. The GPAQ, developed by the World Health Organization, is one of the most commonly used tools for measuring MVPA across not only recreational activities but also work and transportation, as well as sedentary time [47]. However, a limitation of the GPAQ is the potential for recall bias in self-reported measurements, including subjective perceptions of activity intensity and duration [10,47,48]. For this reason, incorporating accelerometers in future studies is recommended.

## 5. Conclusions

Findings from this study suggest that promoting MVPA and strength training while reducing sedentary time can improve cardiometabolic health by increasing skeletal muscle mass while noting that the role of muscle mass as a mediator for MS differs between men and women. This study highlights the importance of skeletal muscle mass in optimizing physical activity for middle-aged and elderly adults to manage their cardiometabolic health. To achieve this, it is recommended to decrease sedentary time, incorporate regular breaks, and engage in 600 MET-minutes of MVPA or more per week, in addition to strength training at least two days per week.

## Figures and Tables

**Table 1:** Participant characteristics.

Variables	Categories	Men			Women		
		Non-MS Group (*n* = 1543)	MS Group (*n* = 1025)	*p*	Non-MS Group (*n* = 2484)	MS Group (*n* = 904)	*p*
Age (years)		60.1 ± 11.7 (59.5–60.7)	60.5 ± 11.1 (59.8–61.1)	0.428	57.8 ± 11.0 (57.3–58.2)	64.3 ± 10.4 (63.6–65.0)	<0.001
Body mass index (kg/m^2^)		23.6 ± 2.5 (23.4–23.7)	26.5 ± 3.1 (26.3–26.7)	<0.001	23.1 ± 2.9 (23.0–23.3)	26.5 ± 3.4 (26.3–26.7)	<0.001
Skeletal muscle mass (% of weight)		32.5 ± 2.6 (32.4–32.6)	30.4 ± 2.2 (30.2–30.5)	<0.001	26.7 ± 2.6 (26.6–26.8)	24.5 ± 2.2 (24.3–24.6)	<0.001
Waist circumference (cm)		85.7 ± 7.2 (85.3–86.0)	94.8 ± 7.3 (94.3–95.2)	<0.001	79.3 ± 8.0 (79.0–79.7)	90.2 ± 7.6 (89.7–90.7)	<0.001
Systolic blood pressure (mmHg)		121.0 ± 14.9 (120.3–121.7)	128.1 ± 15.0 (127.2–129.0)	<0.001	116.8 ± 15.2 (116.2–117.4)	128.2 ± 16.2 (127.1–129.2)	<0.001
Diastolic blood pressure (mmHg)		75.9 ± 9.2 (75.5–76.4)	79.5 ± 10.2 (78.9–80.2)	<0.001	72.9 ± 8.8 (72.6–73.3)	75.6 ± 9.0 (75.1–76.2)	<0.001
Glucose (mg/dL)		100.1 ± 18.7 (99.2–101.0)	116.9 ± 30.4 (115.0–118.7)	<0.001	95.8 ± 14.4 (95.3–96.4)	113.0 ± 28.1 (111.2–114.8)	<0.001
Triglycerides (mg/dL)		113.9 ± 59.3 (110.9–116.8)	208.4 ± 147.0 (199.4–217.4)	<0.001	97.2 ± 42.9 (95.5–98.9)	159.0 ± 93.0 (153.0–165.1)	<0.001
HDL-cholesterol (mg/dL)		55.3 ± 12.8 (54.7–55.9)	45.0 ± 11.4 (44.3–45.7)	<0.001	64.8 ± 14.1 (64.3–65.4)	50.2 ± 12.3 (49.4–51.0)	<0.001
Family history of chronic disease	No/unknown	641 (41.5)	350 (34.1)	<0.001	711 (28.6)	275 (30.4)	0.308
	Yes	902 (58.5)	675 (65.9)		1773 (71.4)	629 (69.6)	
Menopause	No	Not applicable			777 (31.3)	117 (12.9)	<0.001
	Yes	Not applicable			1707 (68.7)	787 (87.1)	
Marital status	Married	1307 (84.7)	829 (80.9)	0.011	1898 (76.4)	596 (65.9)	<0.001
Unmarried		236 (15.3)	196 (19.1)		586 (23.6)	308 (34.1)	
Education High school graduate or lower		869 (56.3)	634 (61.9)	0.005	1592 (64.1)	766 (84.7)	<0.001
College or higher		674 (43.7)	391 (38.1)		892 (35.9)	138 (15.3)	
Household income	<median	618 (40.1)	456 (44.5)	0.026	1075 (43.3)	540 (59.7)	<0.001
	≥median	925 (59.9)	569 (55.5)		1409 (56.7)	364 (40.3)	
Alcohol consumption	Non-drinker	530 (34.3)	321 (31.3)	0.110	1543 (62.1)	652 (72.1)	<0.001
	Drinker	1013 (65.7)	704 (68.7)		941 (37.9)	252 (27.9)	
Smoking	Non-smoker	1128 (73.1)	693 (67.6)	0.003	2402 (96.7)	872 (96.5)	0.733
	Smoker	415 (26.9)	332 (32.4)		82 (3.3)	32 (3.5)	
Sedentary time (hours/day)		8.5 ± 3.5 (8.3–8.7)	8.8 ± 3.5 (8.5–9.0)	0.071	8.2 ± 3.3 (8.0–8.3)	8.9 ± 3.3 (8.7–9.1)	<0.001
Total weekly MVPA (MET-minutes)		986.5 ± 1541.8 (909.5–1063.5)	818.8 ± 1484.2 (727.9–909.8)	0.006	791.9 ± 1136.6 (747.2–836.6)	584.6 ± 920.2 (524.5–644.7)	<0.001
Weekly MVPA < 600 METs		823 (53.3)	610 (59.5)	0.002	1383 (55.7)	609 (67.4)	<0.001
≥600 METs		720 (46.7)	415 (40.5)		1101 (44.3)	295 (32.6)	
Strength training < 2 days/week		977 (63.3)	752 (73.4)	<0.001	2026 (81.6)	786 (86.9)	<0.001
≥2 days/week		566 (36.7)	273 (26.6)		458 (18.4)	118 (13.1)	
Energy intake (Kcal)		2053.5 ± 756.5 (2015.7–2091.3)	2125.0 ± 839.3 (2073.6–2176.4)	0.028	1554.0 ± 604.8 (1530.2–1577.8)	1451.9 ± 560.9 (1415.3–1488.5)	<0.001
Carbohydrate (% of energy intake)		59.7 ± 12.8 (59.0–60.3)	58.7 ± 13.4 (57.8–59.5)	0.054	60.7 ± 12.3 (60.2–61.2)	64.7 ± 11.7 (63.9–65.5)	<0.001
Fat (% of energy intake)		21.5 ± 8.7 (21.1–21.9)	20.9 ± 8.3 (20.4–21.4)	0.092	23.0 ± 9.5 (22.6–23.4)	19.7 ± 8.9 (19.1–20.3)	<0.001
Protein (% of energy intake)		15.1 ± 3.8 (15.1–14.9)	15.0 ± 3.8 (14.8–15.2)	0.469	15.3 ± 4.1 (15.2–15.5)	14.7 ± 3.8 (14.4–14.9)	<0.001

Data are presented as *n* (%) or mean ± standard deviation (95% confidence interval). Abbreviations: HDL, high-density lipoprotein; MS, metabolic syndrome; MVPA, moderate-to-vigorous physical activity; MET, metabolic equivalent of task.

**Table 2:** Pearson correlation coefficient (*r*).

	Age	BMI	SMM	WC	SBP	DBP	Glu	TG	HDLC	NMSC	ST	MVPA
Age	1											
BMI	−0.035 **	1										
SMM	−0.193 ***	−0.361 ***	1									
WC	0.127 ***	0.853 ***	−0.152 ***	1								
SBP	0.328 ***	0.197 ***	−0.094 ***	0.265 ***	1							
DBP	−0.094 ***	0.170 ***	0.084 ***	0.200 ***	0.669 ***	1						
Glu.	0.117 ***	0.174 ***	−0.029 *	0.244 ***	0.149 ***	0.071 ***	1					
TG	−0.075 ***	0.219 ***	0.037 **	0.285 ***	0.149 ***	0.211 ***	0.176 ***	1				
HDLC	−0.091 ***	−0.286 ***	−0.071 ***	−0.391 ***	−0.106 ***	−0.069 ***	−0.174 ***	−0.418 ***	1			
NMSC	0.232 ***	0.538 ***	−0.195 ***	0.636 ***	0.407 ***	0.273 ***	0.422 ***	0.486 ***	−0.510 ***	1		
ST	−0.010	0.060 ***	−0.039 **	0.096 ***	0.003	−0.002	0.024	0.062 ***	−0.063 ***	0.076 ***	1	
MVPA	−0.133 ***	0.021	0.116 ***	−0.020	−0.029 *	0.028 *	−0.037 **	−0.004	0.065 ***	−0.065 ***	−0.129 ***	1

Abbreviations: BMI, body mass index; SMM, skeletal muscle mass; WC, waist circumference; SBP, systolic blood pressure; DBP, diastolic blood pressure; Glu, glucose; TG, triglycerides; HDLC, high-density lipoprotein cholesterol; NMSC, number of metabolic syndrome criteria; ST, sedentary time; MVPA, moderate-to-vigorous physical activity. * *p* < 0.05, ** *p* < 0.01, *** *p* < 0.001. *n* = 5956.

**Table 3:** Mediating effect of skeletal muscle mass on the association of sedentary time and physical activity with cardiometabolic abnormalities and metabolic syndrome.

Group	X	M	Y	Direct Effect of X on Y (c’)	Indirect Effect of X on Y (a × b)	95% CI of Indirect Effect		Mediation of M
						Lower Bound	Upper Bound	
Men (*n* = 2568)	Sedentary time	Skeletal muscle mass	Cardiometabolic abnormalities (MS criteria ≥ 1)	0.021	0.007	0.003	0.013	Full
	MVPA			−0.195	−0.066	−0.110	−0.033	Full
	Strength training			−0.250	−0.074	−0.118	−0.036	Full
	Sedentary time	Skeletal muscle mass	MS (MS criteria ≥ 3)	0.006	0.007	0.003	0.011	Full
	MVPA			−0.143	−0.060	−0.095	−0.032	Full
	Strength training			−0.399 ***	−0.065	−0.100	−0.035	Partial
Women (*n* = 3388)	Sedentary time	Skeletal muscle mass	Cardiometabolic abnormalities (MS criteria ≥ 1)	0.011	0.003	0.001	0.006	Full
	MVPA			−0.125	−0.014	−0.031	−0.002	Full
	Strength training			−0.346 **	−0.023	−0.050	−0.003	Partial
	Sedentary time	Skeletal muscle mass	MS (MS criteria ≥ 3)	0.057 ***	0.001	−0.001	0.004	No
	MVPA			−0.222 *	−0.007	−0.022	0.004	No
	Strength training			−0.173	−0.013	−0.037	0.007	No

Abbreviations: X, independent variable; M, mediator; Y, dependent variable; CI, confidence interval; MS, metabolic syndrome; MVPA, moderate-to-vigorous physical activity. Covariates: age, body mass index, education level, marital status, household income, smoking, daily total energy intake, the proportion of daily energy from protein, fat, and carbohydrate, and menopausal status for women. * *p* < 0.05, ** *p* < 0.01, *** *p* < 0.001.

## Data Availability

The raw data from the 2022 and 2023 KNHANES used in this study are publicly available at https://knhanes.kdca.go.kr/knhanes/main.do (accessed 1 June 2025).

## Abbreviations

The following abbreviations are used in this manuscript:

BIA	Bioelectrical Impedance Analysis
BMI	Body Mass Index
GPAQ	Global Physical Activity Questionnaire
HDL	High-Density Lipoprotein
KNHANES	Korean National Health and Nutrition Examination Survey
MET	Metabolic Equivalent of Task
MS	Metabolic Syndrome
MVPA	Moderate-to-Vigorous Physical Activity
